# Violent Crime, Epilepsy, and Traumatic Brain Injury

**DOI:** 10.1371/journal.pmed.1001148

**Published:** 2011-12-27

**Authors:** Jan Volavka

**Affiliations:** Professor Emeritus of Psychiatry, New York University School of Medicine, New York, New York, United States of America

## Abstract

Jan Volavka discusses new research by Seena Fazel and colleagues that reports increased risk for violent crime among people with traumatic brain injury and epilepsy.

**Linked Research Article**

This Perspective discusses the following new study published in *PLoS Medicine*:

Fazel S, Lichtenstein P, Grann M, Långström N (2011) Risk of Violent Crime in Individuals with Epilepsy and Traumatic Brain Injury: A 35-Year Swedish Population Study. PLoS Med 8(12): e1001150. doi:10.1371/journal.pmed.1001150

Seena Fazel and colleagues report findings from a longitudinal follow-up study in Sweden that evaluated the risks of violent crime subsequent to hospitalization for epilepsy, or traumatic brain injury. The researchers control for familial confounding with sibling controls. The analyses call into question an association between epilepsy and violent crime, although they do suggest that there may be a relationship between traumatic brain injury and violent crime.

In this week's *PLoS Medicine*, Seena Fazel and colleagues report elevated risks for violent crime among patients with traumatic brain injury (TBI) as well as among those with epilepsy, compared to general population controls [1]. Among the major strengths of the study are the very large sample size, comprising the entire population of Sweden, and the follow-up of 35 years. The findings are of major public health importance and provide inspiration for further research.

## Traumatic Brain Injury

The significance of the TBI findings is particularly stark in the United States context. As of 2008, the number of TBIs in the wars in Iraq and Afghanistan was estimated to be at least 320,000 [2]. At the same time, reports have revealed veterans charged with homicides committed after their return home [3]. Homicides by returning veterans have been linked to post-traumatic stress disorder (PTSD) and/or TBI, and this link to the combat-related conditions has led some forensic psychiatrists to propose the enactment of a categorical exclusion of the death penalty for such cases [4]. Other types of violent crime by returning veterans are also frequently reported by the American media [4]. The relative importance of PTSD, TBI, and substance use disorders in the pathway to these crimes is unclear, so the clear demonstration of the link between violent crime and TBI in non-combat cases presented by Fazel and colleagues is thus particularly important. In this context, it is also noteworthy that the authors have adjusted their final analysis for comorbid substance abuse; the result is a reduced, but still statistically significant, odds ratio for risk of violent crime in TBI patients.

One of the unique strengths of the study is that familial confounding was controlled by comparison with unaffected siblings. For TBI, the comparison with unaffected siblings (those without TBI) yielded an odds ratio that was smaller than that obtained with the general population as the comparison group, but still statistically significant.

Familial confounding, reflected by the difference between these two odds ratios, could have been due to genetic or early environmental influences. Both of these domains were explored in a paper by Pardini et al. [5] that can be seen as complementing the present Fazel et al. study. Pardini examined 155 patients with penetrating TBI and 42 controls. All patients were genotyped for the monoamine oxidase A (MAO-A) functional polymorphism yielding low or high transcriptional activity. Assessments of early childhood traumatic experiences as well as current PTSD and aggression were implemented. TBI patients were divided into two groups: those with lesions in the prefrontal cortex (PFC) and those with lesions elsewhere. Patients with PFC lesions were more likely to be aggressive. Lesion location and MAO-A polymorphism interacted in their effects on aggression. Early experiences and current PTSD also had effects on aggression.

The Swedish registers utilized by Fazel and colleagues did not contain usable data on comorbidity with personality disorders, depression, and other psychiatric disorders. This is a limitation; the relationship between antisocial personality disorder (or psychopathy) and violence is clearly important, and a link between depression and aggression in TBI patients has been documented [6]. Furthermore, it would have been useful to examine the data on violent convictions of TBI patients in the context of their violent victimization. Perhaps such data were not available, and future research may be more informative in this respect.

## Epilepsy

In epilepsy, violent behavior may occur during seizures (ictal violence) or in between the seizures (interictal violence). A case report of ictal violence cited by Fazel and colleagues was published by Shih et al., who claimed that the violence was directed and interactive; potential significant adverse legal ramifications of such ictal behavior were mentioned [7]. However, this claim was criticized in a commentary [8]. Similar to most other cases of ictal violence, the patient's movements were stereotyped, resistive, and not targeted to specific individuals. Ictal violence was studied in a series of patients and found to be of very little concern [9]. If epilepsy makes any contribution to violent crime rates, it would be the interictal violence that would be involved.

Victimization of people with epilepsy is a problem. Adult patients are frequently assaulted [10] and children with epilepsy are bullied [11]. Some of the violent crime by people with epilepsy recorded in the Swedish registers may have occurred in response to victimization.

The Fazel study found significantly increased odds of violent crime among epilepsy patients compared with population controls (adjusted odds ratio = 1.5, 95% CI: 1.4–1.7). However, this association disappeared when individuals with epilepsy were compared with their unaffected siblings (adjusted odds ratio = 1.1, 0.9–1.2). This means that the increased risk for violent crime found in comparison with population controls was due to familial confounding. In other words, this increase was due to genetic and/or early environmental influences rather than to epilepsy per se. Previous reports of increased risk of violence in epilepsy did not account for familial confounding, and that literature must now be re-evaluated in the light of this landmark finding. The details of familial effects elevating the risk for violence in epilepsy patients are unclear and future research may address this issue.

Fazel and colleagues extracted data on violent crime convictions that occurred *after* the diagnosis of epilepsy or TBI. Given the strong effects of familial confounding on the risk of violence, it seems that convictions occurring *before* the diagnoses could also be of interest. The confounding presumably affects the risk present before the diagnosis, and studying its effect in isolation (i.e., without the presence of TBI or epilepsy) could be important. Comparing the conviction rates before and after the diagnosis would provide another perspective on the effect of the illness on violent crime. Perhaps this will be done in another study by this group of investigators.

## Abbreviations

PTSD: post-traumatic stress disorder
TBI: traumatic brain injury

## References

[1] Fazel S, Lichtenstein P, Grann M, Langstrom N (2011). Risk of violent crime in individuals with epilepsy and traumatic brain injury: A 35-Year Swedish Population Study.. PLoS Med.

[2] MacDonald CL, Johnson AM, Cooper D, Nelson EC, Werner NJ (2011). Detection of blast-related traumatic brain injury in U.S. military personnel.. N Engl J Med.

[3] Sontag D, Alvarez L (13 January 2008). Across America, deadly echoes of foreign battles.. The New York Times.

[4] Wortzel HS, Arciniegas DB (2010). Combat veterans and the death penalty: a forensic neuropsychiatric perspective.. J Am Acad Psychiatry Law.

[5] Pardini M, Krueger F, Hodgkinson C, Raymont V, Ferrier C (2011). Prefrontal cortex lesions and MAO-A modulate aggression in penetrating traumatic brain injury.. Neurology.

[6] Baguley IJ, Cooper J, Felmingham K (2006). Aggressive behavior following traumatic brain injury: how common is common?. J Head Trauma Rehabil.

[7] Shih JJ, LeslieMazwi T, Falcao G, Van GJ (2009). Directed aggressive behavior in frontal lobe epilepsy: a video-EEG and ictal spect case study.. Neurology.

[8] Fessler AJ, Treiman DM (2009). Epilepsy and aggression: proceed with caution.. Neurology.

[9] Delgado-Escueta AV, Mattson RH, King L, Goldensohn ES, Spiegel H (1981). The nature of aggression during epileptic seizures.. N Engl J Med.

[10] Kwon C, Liu M, Quan H, Thoo V, Wiebe S (2011). Motor vehicle accidents, suicides, and assaults in epilepsy: a population-based study.. Neurology.

[11] Hamiwka LD, Yu CG, Hamiwka LA, Sherman EM, Anderson B, Wirrell E (2009). Are children with epilepsy at greater risk for bullying than their peers?. Epilepsy Behav.

